# Association of the vitamin D metabolite ratio with bone turnover markers and changes in volumetric BMD

**DOI:** 10.1093/jbmrpl/ziag044

**Published:** 2026-03-23

**Authors:** Emma Mulligan, Terri Blackwell, Andrew N Hoofnagle, Olivia Alison Potok, Jane A Cauley, Andrew J Burghardt, Kristine Ensrud, Deborah M Kado, Joachim H Ix, Eric Orwoll, Peggy M Cawthon, Charles Ginsberg

**Affiliations:** Division of Nephrology-Hypertension, University of California, San Diego, San Diego, CA 92093, United States; California Pacific Medical Center Research Institute and Department of Epidemiology and Biostatistics, University of California San Francisco, San Fransisco, CA 94143, United States; Departments of Laboratory Medicine and Pathology and Medicine and the Kidney Research Institute, University of Washington, Seattle, WA 98104, United States; Division of Nephrology-Hypertension, University of California, San Diego, San Diego, CA 92093, United States; Nephrology Section, Veterans Affairs San Diego Healthcare System, San Diego, CA 92161, United States; Department of Epidemiology, School of Public Health, University of Pittsburgh, Pittsburgh, PA 15261, United States; Department of Radiology and Biomedical Imaging, University of California, San Francisco, San Francisco, CA 94143, United States; Center for Care Delivery and Outcomes Research, Minneapolis Veterans Affairs Healthcare System, Minneapolis, MN 55417, United States; Department of Medicine and Division of Epidemiology and Community Health, University of Minnesota, Minneapolis, MN 55455, United States; Department of Medicine, Stanford University, Palo Alto, CA 94305, United States; Geriatric Research Education and Clinical Center (GRECC), Veterans Affairs Healthcare System, Palo Alto, CA 94304, United States; Division of Nephrology-Hypertension, University of California, San Diego, San Diego, CA 92093, United States; Nephrology Section, Veterans Affairs San Diego Healthcare System, San Diego, CA 92161, United States; Division of Endocrinology, Metabolism and Clinical Nutrition, Department of Medicine, Oregon Health and Sciences University, Portland, OR 97239, United States; California Pacific Medical Center Research Institute and Department of Epidemiology and Biostatistics, University of California San Francisco, San Fransisco, CA 94143, United States; Division of Nephrology-Hypertension, University of California, San Diego, San Diego, CA 92093, United States

**Keywords:** vitamin D, bone density, bone turnover, vitamin D metabolite ratio, CTX-I, PINP

## Abstract

The vitamin D metabolite ratio (VMR), calculated as the ratio of 24,25(OH)_2_D_3_ to 25(OH)D, is a marker of vitamin D stores that may provide a more accurate reflection of bone health outcomes than 25(OH)D alone. In this analysis, we examined the relationship between VMR and 25(OH)D with bone turnover markers (BTM’s) and changes in volumetric BMD (Tt.BMD) in older community-dwelling men participating in the Osteoporotic Fractures in Men (MrOS) study. Tt.BMD was measured at the distal radius and tibia by HR-pQCT at 2 separate visits approximately 6 yr apart. Bone turnover markers were measured at the time of the first HR-pQCT scan and included PTH, C-terminal telopeptide of type I collagen (CTX-I), and N-terminal propeptide of type I procollagen (PINP). Linear regression was used to evaluate the association of VMR and 25(OH)D with BTM’s and annualized percent change in Tt.BMD. The mean (SD) age of the 254 men was 83(3) yr, with an eGFR of 71(14) mL/min/1.73 m^2^. Mean VMR and 25(OH)D were 6.5(2.2) (ng/mL)/(ng/mL) and 39(14) ng/mL, respectively. Both the VMR and 25(OH)D were inversely associated with PTH concentrations (*p* < .02 for both). In fully adjusted models, a 2-fold higher VMR was associated with a 34% [12%; 55%] lower CTX-I and a 16% [1.3%;30%] lower PINP while 25(OH)D was not associated with these BTMs. The relationship of VMR with BTM’s appeared stronger among men with CKD (eGFR <60 mL/min/1.73 m^2^) than in persons with normal renal function. Neither VMR nor 25(OH)D were associated with annualized change in Tt.BMD in fully adjusted models.

## Graphical Abstract

**Graphical Abstract:**
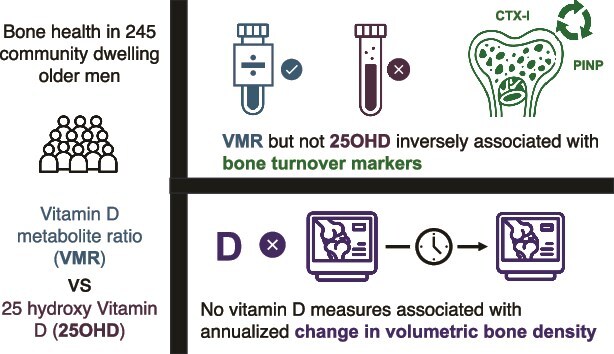

## Introduction

When defined by 25(OH)D concentration levels less than 20 ng/mL, vitamin D deficiency is common across the globe, particularly among elderly persons, nursing home residents, and hospitalized persons.^1^^,^^2^ Vitamin D plays a major role in the regulation of calcium and phosphorous homeostasis,^3^ both components of bone matrix.^4^ Severe vitamin D deficiency can cause rickets and osteomalacia.^2^ However, recent studies examining the association between vitamin D supplementation and bone health have provided conflicting results.^5^ We hypothesized that the utilization of 25(OH)D alone to assess vitamin D status may provide an incomplete picture of vitamin D activity as it relates to bone turnover and changes in bone mass in older community-living individuals.

Currently available laboratory tests may offer an incomplete picture of vitamin D activity. In current clinical practice, 25(OH)D is the most commonly used measure of vitamin D stores. However, it may not be the ideal measure of vitamin D activity, particularly with regards to bone health. 25(OH)D is not the active form of vitamin D and its concentration is dependent on levels of vitamin D binding protein.^6^ Prior studies have shown 25(OH)D has a weak relationship^7^ with indices of bone health with significant variability across races.^8–10^ Other measures of vitamin D have been proposed including the vitamin D metabolite ratio (VMR), the ratio between serum 24,25(OH)_2_D_3_ and 25(OH)D. With sufficient tissue level vitamin D stores, a higher portion of 25(OH)D is catabolized to the inactive form 24,25(OH)_2_D_3_, reflected by a higher VMR. Consequently, VMR (vitamin D metabolite ratio) is expected to be low when tissue-level vitamin D stores are insufficient and high when stores are sufficient. The VMR is independent of vitamin D binding protein levels^6^ and correlates better with other measures of bone turnover across races.^8–10^

Commonly used measures of bone turnover include PTH, C-terminal telopeptide of type I collagen (CTX-I, utilized as a measure of bone resorption), and N-terminal propeptide of type I procollagen (PINP, utilized as a measure of bone formation).^11^ Prior studies^12–14^ conducted in a variety of populations have found an inverse association between VMR, 25(OH)D, and 24,25(OH)_2_D_3_ with PTH. These papers have shown conflicting results for the relationship between vitamin D measures and CTX-I and PINP, with some showing negative association and some showing no association. Since vitamin D sufficiency at the level of bone should reduce bone turnover, we hypothesized that a higher VMR would be associated with lower concentrations of PTH, CTX-I, and PINP in our cohort of community-dwelling older men, and that these associations would be stronger than those between 25(OH)D and these bone turnover markers (BTMs).

In addition to posited effects of vitamin D on BTMs, in our prior work,^15^ we found that a higher VMR was associated with greater total volumetric BMD (Tt.BMD) and bone strength by estimated failure load (FL) from micro-finite element analysis (μFEA) at a single point in time in older men, while 25(OH)D had no such association. Based upon this prior study and the availability of repeated measures of Tt.BMD in this cohort, we further hypothesized that a higher VMR would be associated with preserved Tt.BMD and FL over time.

To test these hypotheses, we used data from the Osteoporotic Fractures in Men (MrOS) study of community-dwelling men to determine the association of VMR and 25(OH)D with BTMs and longitudinal change in Tt.BMD and FL.

## Materials and methods

### Study population

**Figure 1:**
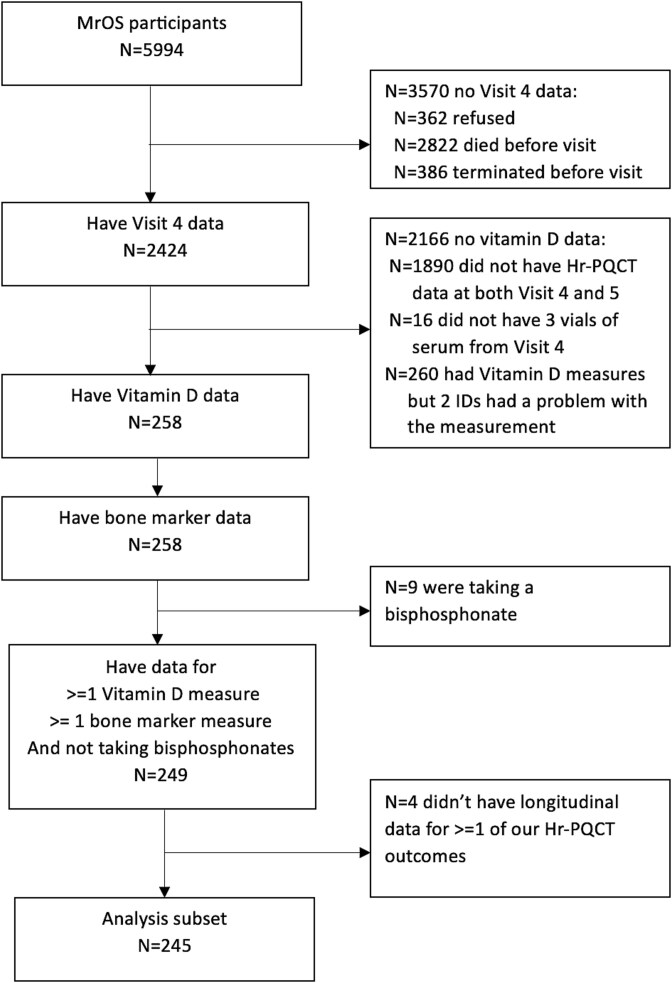
Participant selection.

The MrOS study is a longitudinal, observational cohort of community dwelling older men designed to evaluate risk factors for osteoporosis and fractures.^16^^,^^17^ Between March 2000 and April 2002, 5994 men aged 65 or older were recruited from 6 centers across the United States (Birmingham, Alabama; Minneapolis, Minnesota; Palo Alto, California; Monongahela Valley near Pittsburgh, Pennsylvania; Portland, Oregon; and San Diego, California). The study was approved by the central and/or local institutional review boards and informed consent was obtained from all MrOS participants. From May 2014 to May 2016, all active participants were invited to participate in MrOS “Visit 4,” with 2424 men completing some part of the visit. Between August 2020 and May 2022, active participants from 3 sites (Palo Alto, California; Pittsburgh, Pennsylvania; and Portland, Oregon) who had a good quality HR-pQCT image at Visit 4 were invited to participate in MrOS Visit 5. The analytical cohort for this study was comprised of 245 men who were not taking bisphosphonates and had data for the vitamin D metabolites at visit 4, HR-pQCT data at both Visit 4 and Visit 5, and BTMs at visit 4 (Figure 1).

### Exposure variables

Vitamin D metabolites 25(OH)D_2_, 25(OH)D_3_, 24,25(OH)_2_D_3_, and 1,25(OH)_2_D were tested from stored blood samples collected at Visit 4 in men who had HR-pQCT measurements at Visits 4 and 5 and had 3 or more vials of stored serum. Samples were stored at −80 °C from collection until testing. All the aforementioned metabolites were measured using liquid–liquid extraction and subsequent liquid chromatography-tandem mass spectrometry, as described previously.^15^ The VMR was calculated by dividing serum 24,25(OH)_2_D_3_ by serum 25(OH)D_3_ and multiplying by 100. The total 25(OH)D was calculated by the sum of 25(OH)D_2_ and 25(OH)D_3_.

### Outcome variables

The primary outcomes for this analysis were PTH, CTX-I, PINP, and the percent annualized change in total Tt.BMD and FL of the distal radius and tibia. PTH, CTX-I, and PINP were selected as BTMs. These markers were measured at Visit 4. PTH was measured using an automated sandwich immunoassay on a Beckman DxI 800. The concentration of intact PINP and the degradation products of type 1 collagen (including CTX-I) were quantified by 2 different automated immunochemical methods on the same immunodiagnostic systems (IDS) iSYS platform according to the manufacturer’s instructions. These are 2-site sandwich chemiluminometric immunoassays.

Total BMD and FL were measured at Visits 4 and 5 from HR-pQCT scans of the distal radius and tibia metaphyses, positioned 9 and 22 mm proximal to their respective distal articular surfaces.^18^ Tt.BMD calculated at each timepoint in co-registered volumes of interest identified using 3D image registration.^19^ To preserve parallel surfaces suitable for defining appropriate FEA boundary conditions, FL was calculated in a co-registered volumes of interest based on the default 2D registration method, which matches slices according to the total cross-sectional area.^20^ Failure load was computed by linear μFEA. An iterative solver (Scanco FE Software v1.12, Scanco Medical) was used to compute reaction forces at the superior and inferior ends of the sections for a 1% axial compressive strain, assuming a homogeneous tissue modulus (10 GPa) for all bone elements. Failure load was estimated by calculation of the reaction force at which 5% of bone elements exceeded a local effective strain of 1.0%.^21^ The model computations were performed at the UCSF Wynton HPC Co-Op cluster. We calculated the annualized percent change in Tt.BMD as the difference between Tt.BMD at Visit 5 and Visit 4 divided by the Tt.BMD at Visit 4 and divided by the number of years between Visit 4 and Visit 5 multiplied by 100. Percent annualized change in FL was calculated in the same manner.

### Other measurements

All participants self-reported age, race, history of diabetes, activity (calculated by physical activity score for the elderly (PASE)^22^), and smoking status. Blood pressure, weight, and height were measured at clinic visits, and BMI was calculated in kg/m^2^. The clinic location and season of blood draw was recorded at study Visit 4. Serum creatinine values were measured using Visit 4 blood samples. Serum creatinine was quantified with a Beckman AU5812 automated instrument using the Jaffe rate method, which monitors the formation of a colored complex of creatinine and picric acid. The calibration was based on isotope dilution-mass spectrometry. Estimated glomerular filtration rate (eGFR) was calculated using the 2021 CKD epidemiology collaboration creatinine equation.^23^ CKD was defined as eGFR <60 mL/min/1.73 m^2^.^24^ All prescription and nonprescription medications used within 30 d prior to Visit 4 were entered into an electronic database; each medication was matched to its ingredient(s) based on the Iowa Drug Information Service Drug Vocabulary (College of Pharmacy, University of Iowa).^25^ Use of medications containing vitamin D, use of bisphosphonates, and testosterone supplementation were determined. No patients reported use of other medications used in the treatment of osteoporosis.

### Statistical methods

We evaluated the baseline characteristics of participants by VMR quartile utilizing χ^2^ tests for categorical variables, analysis of variance for normally distributed continuous variables, and Kruskal–Wallis tests for skewed continuous variables. We then evaluated the cross-sectional relationship between VMR with PTH, CTX-I and PINP, using multivariable linear regression. To facilitate statistical analysis, all predictor and outcome variables were log transformed such that a 100^*^beta coefficient can be interpreted as a percent increase in outcome variable per 100% increase in predictor variable. We developed 2 models for this relationship: model 1 adjusted for age, self-reported race, season, and clinic location, and model 2 adjusted for model 1 factors as well as BMI, physical activity, smoking status, systolic blood pressure, eGFR, history of diabetes mellitus, and medications containing vitamin D. We created identical models with VMR, 25(OH)D, 24,25(OH)_2_D_3_, and 1,25(OH)_2_D as the independent variables. We then ran identical models in which we evaluated the association of VMR and vitamin D metabolites with longitudinal annualized percent change in Tt.BMD and FL assessed by HR-pQCT at Visits 4 and 5. We created multiplicative interaction terms of the predictor variables with race (White vs non-White) and eGFR (as a continuous variable and as a dichotomous variable with eGFR <60 and eGFR ≥60) which were included separately in models that were adjusted for covariates in model 2. If significant interactions were detected, we subsequently conducted a subset analysis stratified by race or CKD. All significance levels reported were 2-sided and all analyses were conducted using SAS version 9.4 (SAS Institute Inc.).

## Results

The mean age of the 245 participants was 83 yr old and 89% were White. Eleven percent of the participants reported a history of diabetes, and 23% had CKD. The mean ± SD 25(OH)D was 39.3 ± 14.0 ng/mL, 1,25(OH)_2_D was 47.3 ± 13.1 pg/mL, 24,25(OH)2D was 2.6 ± 1.5 ng/mL, and VMR was 6.5 ± 2.2. The mean ± SD PTH was 49.9 ± 30.5 pg/dL, CTX-I was 0.291 ± 0.179 ng/mL, and PINP was 51.0 ± 22.5 ng/mL. The average follow-up time between Visit 4 and 5 was 6.0 ± 0.6 yr. Over this follow-up, the annualized percent change in Tt.BMD was −0.8% ± 1.0% at the distal radius and −0.8% ± 0.8% at the distal tibia. The annualized percent change in FL −1.2% ± 1.7% at the distal radius and −1.1% ± 1.4% at the distal tibia. Baseline characteristics by VMR quartile are shown in Table 1. Compared with persons in the lowest VMR quartile, persons in the highest VMR quartile were more likely to be taking a medication containing vitamin D and were less likely to have CKD.

**Table 1:** Baseline characteristics by vitamin D metabolite ratio quartiles.

	**All**	**Q1: 0.6 to <5.3**	**Q2: 5.3 to <6.5**	**Q3: 6.5 to <7.8**	**Q4: 7.8 to 13.2**	***p*-value**
**Characteristic**	**(*N* = 245)**	**(*N* = 61)**	(*N* = 61)	**(*N* = 61)**	**(*N* = 62)**	
**Age (yr)**	83.0 ± 3.3	83.5 ± 3.4	83.2 ± 3.7	82.5 ± 2.7	82.7 ± 3.2	.2691
**Race**						
** White**	217 (88.6)	53 (86.9)	53 (86.9)	55 (90.2)	56 (90.3)	.4594
** Black**	5 (2.0)	1 (1.6)	3 (4.9)	0	1 (1.6)	
** Asian**	12 (4.9)	6 (9.8)	1 (1.6)	3 (4.9)	2 (3.2)	
** Hispanic**	9 (3.7)	1 (1.6)	3 (4.9)	2 (3.3)	3 (4.8)	
** Other**	2 (0.8)	0	1 (1.6)	1 (1.6)	0	
**Season of blood draw**						
** Winter**	39 (15.9)	13 (21.3)	12 (19.7)	8 (13.1)	6 (9.7)	.1195
** Spring**	59 (24.1)	14 (23.0)	13 (21.3)	15 (24.6)	17 (27.4)	
** Summer**	83 (33.9)	12 (19.7)	21 (34.4)	22 (36.1)	28 (45.2)	
** Fall**	64 (26.1)	22 (36.1)	15 (24.6)	16 (26.2)	11 (17.7)	
**Site**						
** Palo Alto**	60 (24.5)	18 (29.5)	14 (23.0)	18 (29.5)	10 (16.1)	.4359
** Pittsburgh**	86 (35.1)	23 (37.7)	23 (37.7)	17 (27.9)	23 (37.1)	
** Portland**	99 (40.4)	20 (32.8)	24 (39.3)	26 (42.6)	29 (46.8)	
**SBP (mmHg)**	131.6 ± 17.6	131.4 ± 19.1	134.6 ± 17.3	130.0 ± 16.4	130.3 ± 17.7	.4604
**Current or past smoker**	133 (54.3)	32 (52.5)	35 (57.4)	30 (49.2)	36 (58.1)	.7257
**History of diabetes mellitus**	26 (10.6)	7 (11.5)	8 (13.1)	4 (6.6)	7 (11.3)	.6735
**eGFR (mL/min/1.73 m^2^)**	70.9 ± 14.4	63.1 ± 16.1	69.8 ± 14.9	75.4 ± 11.5	75.1 ± 11.0	<.0001
**CKD: eGFR <60 mL/min/1.73 m^2^**	56 (22.9)	27 (44.3)	19 (31.1)	5 (8.2)	5 (8.1)	<.0001
**BMI (kg/m^2^)**	26.9 ± 3.3	27.6 ± 3.2	27.4 ± 3.5	26.3 ± 3.5	26.3 ± 3.1	.0566
**PASE physical activity score**	139.0 ± 67.3	137.9 ± 72.9	134.8 ± 64.8	147.0 ± 69.6	136.0 ± 62.4	.7460
**Taking medication containing Vitamin D**	185 (75.5)	33 (54.1)	43 (70.5)	53 (86.9)	56 (90.3)	<.0001
**25 (OH)D (ng/mL)**	39.3 ± 14.0	33.2 ± 14.6	38.9 ± 11.0	41.0 ± 12.4	44.1 ± 15.5	.0001
**24,25 (OH)_2_D_3_(ng/ml)**	2.6 ± 1.5	1.2 ± 0.7	2.2 ± 0.7	2.9 ± 0.8	4.0 ± 1.9	<.0001
**1,25 (OH)_2_D (pg/mL)**	47.3 ± 13.1	46.8 ± 14.2	47.1 ± 13.6	47.8 ± 10.7	47.4 ± 14.0	.9762
**PTH (pg/mL)**	49.9 ± 30.5	67.2 ± 49.1	50.5 ± 18.8	42.5 ± 18.0	39.7 ± 14.9	<.0001
**CTX-I (ng/mL)**	0.291 ± 0.179	0.326 ± 0.158	0.273 ± 0.163	0.273 ± 0.172	0.293 ± 0.216	0.0800
**PINP (ng/mL)**	51.0 ± 22.5	53.8 ± 20.5	46.9 ± 15.6	50.4 ± 27.0	52.8 ± 25.1	.3095
**Annualized percent change in distal radius BMD, mg/cm^3^**	−0.8 ± 1.0	−0.8 ± 1.0	−0.8 ± 1.1	−0.7 ± 0.7	−0.9 ± 1.1	.8030
**Annualized percent change in distal tibia BMD, mg/cm^3^**	−0.8 ± 0.8	−0.8 ± 0.9	−0.8 ± 0.8	−0.6 ± 0.6	−0.8 ± 0.9	.5277
**Annualized percent change in distal radius failure load, *N***	−1.2 ± 1.7	−1.4 ± 1.8	−1.2 ± 1.9	−1.1 ± 1.5	−1.3 ± 1.8	.8506
**Annualized percent change in distal tibia estimated failure load, *N***	−1.1 ± 1.4	−1.3 ± 1.6	−1.1 ± 1.4	−0.9 ± 0.9	−1.1 ± 1.6	.5879
**Follow-up time visit 4 to visit 5 (yr)**	6.0 ± 0.6	6.1 ± 0.6	6.0 ± 0.6	6.0 ± 0.6	6.0 ± 0.5	.5131

Data shown as mean ± SD or *n* (%).

*p*-values testing for differences across quartiles. *p*-values for continuous variables are from ANOVA for normally distributed variables or from a Kruskal–Wallis test for skewed data. *p*-values for categorical data are from a chi-square test for homogeneity.

Abbreviations: CKD, chronic kidney disease (eGFR <60 mL/min/1.73 m^2^); CTX-I, C-terminal telopeptide of type I collagen; eGFR, estimated glomerular filtration rate; PINP, N-terminal propeptide of type I procollagen; SBP, systolic blood pressure.

In our fully adjusted model, a 2-fold higher VMR was associated with a 42% (95% CI: 28%, 57%) lower PTH (Table 2). 25(OH)D and 24,25(OH)_2_D_3_ were also inversely associated (albeit numerically weaker) with PTH [−22% (−40%, −4.1%) and −23% (−32%, −14%), respectively]. There was no significant association of 1,25(OH)_2_D with PTH. VMR was inversely associated with CTX-I such that a 2-fold higher VMR was associated with a 34% (12%, 55%) lower CTX-I. A 2-fold higher 24,25(OH)2D3 was associated with a 14% (0.7%, 27%) lower CTX-I. A 2-fold higher 1,25(OH)_2_D was associated with a 60% (33%, 87%) higher CTX-I. There were no associations between 25(OH)D and CTX-I (*p* = .7226). In the fully adjusted model, a 2-fold higher VMR was associated with a 16% (1.3%, 30%) lower PINP. None of the vitamin D metabolites studied showed a significant association with PINP (*p* > .05 for all).

**Table 2:** Association of vitamin D metabolites and the VMR with markers of bone turnover.^*^

	**PTH**		**CTX-I**		**PINP**	
	**Percent higher PTH per 2-fold increase (95% CI)**	***p*-value**	**Percent higher CTX-I per 2-fold increase (95% CI)**	***p*-value**	**Percent higher PINP per percent higher (95% CI)**	***p*-value**
**VMR**						
**Model 1**	−51.3 (−63.4, −39.2)	<.0001	−23.5 (−42.0, −5.1)	.0131	−7.9 (−20.0, 4.1)	.1991
**Model 2**	−42.0 (−56.5, −27.5)	<.0001	−33.5 (−54.8, −12.3)	.0022	−15.8 (−30.4, −1.3)	.0342
**25(OH)D**						
**Model 1**	−24.1 (−39.8, −8.4)	.0030	8.4 (−13.4, 30.2)	.4495	−1.7 (−15.7, 12.4)	.8171
**Model 2**	−22.1 (−40.1, −4.1)	.0168	−4.6 (−30.1, 20.9)	.7226	−8.4 (−25.7, 8.9)	.3430
**24,25(OH)_2_D_3_**						
**Model 1**	−27.7 (−35.4, −19.9)	<.0001	−6.8 (−18.4, 4.8)	.2524	−3.3 (−10.8, 4.1)	.3830
**Model 2**	−23.0 (−32.1, −13.8)	<.0001	−14.1 (−27.4, −0.7)	.0396	−8.0 (−17.0, 1.1)	.0857
**1,25(OH)_2_D**						
**Model 1**	5.1 (−15.0, 25.1)	.6213	48.0 (21.2, 74.7)	.0005	13.5 (−4.1, 3.1)	.1336
**Model 2**	10.7 (−9.6, 31.0)	.3019	60.0 (32.5, 87.4)	<.0001	18.6 (−0.6, 37.8)	.0582

Model 1: Adjusted for age, race (White vs non-White), clinic, and season of blood draw.

Model 2: Model 1 + physical activity, BMI, smoking status (ever vs never), self-reported history of diabetes mellitus, systolic blood pressure, estimated glomerular filtration rate, and use of medication containing Vitamin D.

Abbreviations: CTX-I, C-terminal telopeptide of type I collagen; PINP, N-terminal propeptide of type I procollagen; VMR, vitamin D metabolite ratio. ^*^ Reported as 100^*^beta coefficient (95% confidence interval), modeled with both outcome and predictor log-transformed.

**Figure 2:**
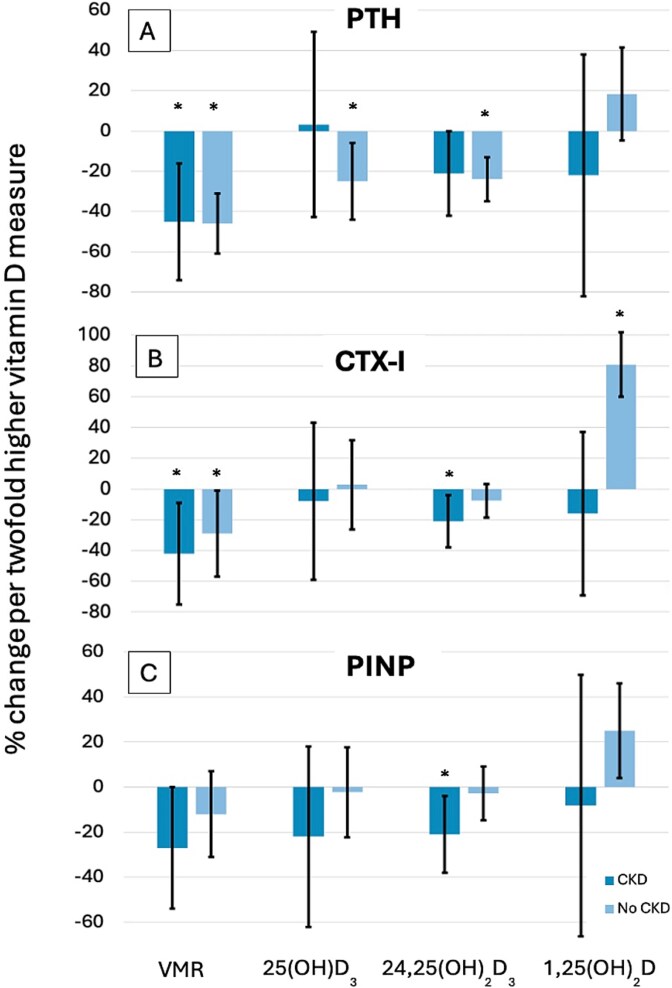
Association of vitamin D metabolites and the VMR with PTH (A), CTX-I (B), and PINP (C) in patients with and without CKD. ^*^p < .05. Model adjusted for age, race (White vs non-White), clinic, season of blood draw, physical activity (PASE), BMI, smoking status (ever vs never), self-reported history of diabetes mellitus, systolic blood pressure, and use of medication containing vitamin D. VMR, vitamin D metabolite ratio; CTX-I, C-terminal telopeptide of type I collagen; PINP, N-terminal propeptide of type I procollagen; eGFR-CKD, CKD (estimated glomerular filtration rate <60 mL/min/1.73 m^2^).

There was a significant interaction for the relationship of 1,25(OH)_2_D with eGFR on PTH and CTX-I (*p* = .0021 and .0045, respectively). Based upon this interaction, we conducted a stratified analysis of persons with [*n* = 56 (23%)] and without [*n* = 189 (77%)] CKD (Figure 2, Table S1). Within the subset of persons without CKD, for every 2-fold higher VMR, there was a 29% lower (1.9%, 57%) CTX-I. For persons without CKD, every 2-fold higher 1,25(OH)_2_D was associated with an 80.9% higher (49%, 112%) CTX-I while there was no association between 1,25(OH)_2_D and CTX-I in persons with CKD. Within the subset of persons with CKD, for every 2-fold higher 24,25(OH)_2_D_3_, there was a 21% lower (2.9%, 39%) PINP. Among the CKD subset, the VMR was not associated with PINP [−27% (−55%, 0.5%), *p* = .054]. Among persons without CKD, for every 2-fold higher 1,25(OH)_2_D, there was a 25% higher (2.8%, 46%) PINP.

We did not find a statistically significant association between any measure of vitamin D and annualized percent change in Tt.BMD at the distal radius nor distal tibia sites (*p* > .1958 for all measurements Table 3). Results for annualized percent changes in FL at both sites were similarly non-significant (*p* > .0547 for all measurements, see Table 3). There was no evidence of interaction between the vitamin D metabolites and race or eGFR on changes in Tt.BMD or FL.

**Table 3:** Association of vitamin D metabolites and the VMR with annualized percent change in volumetric BMD and annualized percent change in estimated fracture load at the distal radius and distal tibia.

	**Radius Tt.BMD**		**Tibia Tt.BMD**		**Radius FL**		**Tibia FL**	
	**Percent higher Tt.BMD per 2-fold increase (95% CI)**	***p*-value**	**Percent higher Tt.BMD per percent higher (95% CI)**	***p*-value**	**Percent higher FL per percent higher (95%CI)**	***p*-value**	**Percent higher FL per 2-fold increase (95% CI)**	***p*-value**
**VMR**								
**Model 1**	0.1 (−7.3, 7.5)	.9766	0.6 (−5.8, 6.9)	.8606	3.1 (−6.9, 13.1)	0.5408	0.9 (−7.6, 9.5)	.8297
**Model 2**	3.7 (−5.4, 12.7)	.4298	4.6 (−3.2,12.3)	.2517	10.9 (−1.3, 23.1)	0.0826	2.1 (−8.3, 12.6)	.6902
**25(OH)D**								
**Model 1**	3.8 (−4.9, 12.4)	.3948	3.5 (−4.0, 11.0)	.3639	7.6 (−4.0, 19.2)	0.2007	7.4 (−2.7, 17.4)	.1507
**Model 2**	3.3 (−7.5, 14.1)	.5509	3.8 (−5.4, 13.0)	.4201	10.4 (−4.1, 24.9)	0.1625	4.0 (−8.4, 16.3)	.5302
**24,25(OH)_2_D_3_**								
**Model 1**	1.3 (−3.2, 5.9)	.5712	1.2 (−2.8, 5.1)	.5621	3.5 (−2.6, 9.7)	0.2629	2.5 (−2.9, 7.8)	.3683
**Model 2**	2.6 (−3.0, 8.2)	.3671	2.9 (−2.0, 7.7)	.2482	7.4 (−0.1, 15.0)	0.0547	2.0 (−4.5, 8.5)	.5470
**1,25(OH)_2_D**								
**Model 1**	4.9 (−6.8, 16.7)	.4121	−0.7 (−9.9, 8.5)	.8795	−1.4 (−16.9, 14.2)	0.8650	2.5 (−9.9, 14.9)	.6947
**Model 2**	8.4 (−4.3, 21.0)	.1958	1.9 (−8.3, 12.0)	.7174	2.6 (−14.3, 19.6)	0.7606	7.1 (−6.4, 20.6)	.3043

Model 1: Adjusted for age, race (White vs non-White), clinic, and season of blood draw.

Model 2: Model 1 + physical activity, BMI, smoking status (ever vs never), self-reported history of diabetes mellitus, systolic blood pressure, estimated glomerular filtration rate, and use of medication containing Vitamin D.

Abbreviations: FL, failure load; Tt.BMD, volumetric BMD; VMR, vitamin D metabolite ratio.

## Discussion

We evaluated the relationship of multiple different vitamin D metabolites with BTM’s and the annualized percent change in Tt.BMD and FL among 245 community-living older men. We found that the VMR was inversely associated with BTM’s (CTX-I and PINP) in older men, while 25(OH)D had no association. Both VMR and 25(OH)D were inversely associated with PTH. These associations were generally stronger in persons with CKD than in persons with normal renal function. We found that none of the vitamin D metabolites nor VMR were associated with the annualized percent change in Tt.BMD or FL in this same population.

PTH increases bone resorption and turnover^26^^,^^27^ and calcitriol has negative feedback on PTH production.^28^ In prior studies, 25(OH)D and VMR have been inversely associated with PTH.^13^^,^^29^ We found this same relationship in our study in persons with and without CKD. However, the associations between VMR and 25(OH)D with CTX-I and PINP have been inconsistent in prior studies.^13^^,^^14^ A study by Zelzer et al. focused on 131 postmenopausal women in predominantly the fifth and sixth decade of life, half of whom had low BMD and half with normal BMD, and found no associations between vitamin D measures and BMD. A study by Herrmann et al. combined 2 pre-existing cohorts, one of healthy adult blood donors in Austria and one of patients hospitalized for elective diagnostic coronary angiography at a single German hospital. Herrmann et al. created a combined “vitamin D activity factor” that utilized VMR and found an inverse association between low “vitamin D activity factor” and CTX-I. In our population of community-dwelling older men, we found that VMR was inversely associated with PTH, CTX-I, and PINP in fully adjusted models. 1,25(OH)_2_D was directly associated with CTX-I. The positive association between 1,25(OH)_2_D and bone resorption marker CTX-I may be due to the stimulation of osteocalstogenesis and osteoclast differentiation^30^ by 1,25(OH)_2_D. Furthermore, vitamin D exerts dose-dependent effects on the overall balance of bone resorption.^31^^,^^32^ Alternatively, PTH could be increasing vitamin D activation to 1,25(OH)_2_D and bone resorption concurrently.

Based upon the significant interaction between vitamin D metabolites and eGFR as a continuous variable, we conducted a stratified analysis on our data for patients with and without CKD. The patterns and strength of association we found with various vitamin D markers and BTMs differed by CKD status. For persons with CKD, 24,25(OH)_2_D_3_ was inversely associated with CTX-I and PINP. These associations were not seen in the non-CKD subset. This suggests 24,25(OH)_2_D_3_ may be more strongly associated with BTMs in patients with CKD vs normal renal function. The majority of 25(OH)D hydroxylation to active 1,25(OH)_2_D occurs in the renal tubules.^33^ Enzyme levels, enzyme availability, and delivery of 25(OH)D are altered in patients with CKD leading to decreased levels of 1,25(OH)_2_D.^34^ A prior study of bone metabolism and vitamin D supplementation in healthy older adults also found significant differences in multiple bone health measures between persons with and without CKD.^35^ Prior studies have also shown baseline elevated levels of PTH and CTX-I in patients with CKD that decreased after treatment with calcitriol^36^ and cholecalciferol.^37^ In addition, bone turnover is more altered in patients with CKD. CTX-I and total PINP are renally cleared,^38^ which could enhance detection of a signal between biomarkers in these patients. Taken together with our findings, this suggests that vitamin D metabolites may play different roles or have different clinical significance in people with and without CKD.

Prior studies have shown a positive association between VMR and measures of bone density and strength.^15^^,^^39^ In our prior work^15^ in a similar but distinct subset of the MrOS cohort, we found an association between VMR with Tt.BMD and FL in a cross-sectional analysis. We have also previously reported that a higher VMR was associated with a decrease in BMD loss by DXA in older participants of the Health Aging and Body Composition Study.^39^ In this current study, we did not find an association between annualized VMR or vitamin D metabolites and annualized change in Tt.BMD. This may be due to smaller sample size. Our prior MrOS study included 545 studied cross-sectionally at Visit 4 but only 245 of the MrOS study participants survived to Visit 5, were able to attend Visit 5, and had longitudinal HR-pQCT and vitamin D/BTM data required for the present study. There is also a survivorship bias in our cohort as only the healthiest individuals survived and were able to come in person to Visit 5. Tt.BMD was only measured at 2 points in this study, while in Health ABC, there were multiple measurements at more frequent intervals in a younger (70-79 yr old) cohort. Also, our cohort of patients is 89% White and does not represent the diverse populations for whom VMR may be a better measure of vitamin D related bone health. The majority (75.5%) of participants in the study were taking vitamin D containing medications at Visit 4 which limits the generalizability of the study.

This analysis has several strengths. First, MrOS has long-term follow-up and robust data with extensive vitamin D phenotyping, Tt.BMD at 2 visits, and multiple BTMs. In this study, we were able to evaluate these relationships in a unique cohort of men in the ninth and 10th decades of life. This study has important limitations as well. The analysis is of a relatively small and homogenous sample, with community-dwelling, male, predominantly White older adults. Given the sample size, we were underpowered to evaluate for fractures. Additionally, the study is entirely observational in nature, and thus, it remains unclear if interventional studies targeting vitamin D metabolites or the VMR would either lower bone turnover or change Tt.BMD. There is also a survivorship and selection bias as only individuals alive and able/willing to come in for Visit 5 20 yr after initial recruitment are included.

In conclusion, in this cohort of older, community-dwelling men, the VMR but not 25(OH)D was inversely associated with BTM CTX-I and PINP. Both VMR and 25(OH)D were inversely associated with PTH. These relationships appeared stronger among men with CKD than in men with normal renal function. There were no statistically significant associations between vitamin D measures and annualized change in Tt.BMD or FL. Future directions for research include evaluating the relationship of the VMR and 25(OH)D with BTM in a more diverse population and in a larger populations of persons with CKD. Additionally, studies of larger samples may be needed to evaluate the relationship of the VMR with longitudinal changes in bone structure and volume.

## Supplementary Material

Supplementary_Material_ziag044

## Data Availability

Data available upon request from the MrOS Steering Committee (https://mrosonline.ucsf.edu).
