# Supplementary material for: Association of the vitamin D metabolite ratio with bone turnover markers and changes in volumetric BMD
Source: JBMR Plus. 2026 Mar 23;10(5):ziag044. doi: 10.1093/jbmrpl/ziag044 (PMC13089526; doi:10.1093/jbmrpl/ziag044)
Supplement: Supplementary_Material_ziag044 [file supplementary_material_ziag044.docx]

**Table S1 Association of vitamin D metabolites and the VMR with markers of bone turnover for persons with (n=56) and without CKD (n=189).**

|  | PTH | | CTX-I | | PINP | |
| --- | --- | --- | --- | --- | --- | --- |
|  | Percent higher PTH per twofold increase (95% CI) | p-value | Percent higher CTX-I per twofold increase (95% CI) | p-value | Percent higher PINP per percent higher (95% CI) | p-value |
| VMR |  |  |  |  |  |  |
| CKD | - 44.8 (-75.2, -14.4) | 0.0049 | - 42.4 (-76.2, -8.7) | 0.0151 | - 27.1 (-54.6, 0.5) | 0.0543 |
| No CKD | - 42.3 (-59.6, -25.0) | <0.0001 | - 29.2 (-56.5, -1.9) | 0.0373 | -12.4 (-30.5, 5.6) | 0.1787 |
| p-interaction |  | 0.7049 |  | 0.5890 |  | 0.8903 |
| 25(OH)D |  |  |  |  |  |  |
| CKD | 3.2 (-44.8, 51.2) | 0.8929 | - 8.0 (-61.0, 45.0) | 0.7618 | - 22.3 (-62.9, 18.4) | 0.2752 |
| No CKD | - 24.8 (-44.1, -5.4) | 0.0130 | 2.8 (-26.8, 32.4) | 0.8540 | -2.3 (-21.7, 17.1) | 0.8165 |
| p-interaction |  | 0.3184 |  | 0.4285 |  | 0.4604 |
| 24,25(OH)_2_D_3_ |  |  |  |  |  |  |
| CKD | - 21.2 (-42.3, -0.8) | 0.0491 | -25.9 (-48.6, -3.2) | 0.0262 | -20.8 (-38.7, -2.9) | 0.0236 |
| No CKD | - 23.9 (-34.5, -13.2) | <0.0001 | -7.7 (-24.5, 9.1) | 0.3705 | -2.8 (-13.8, 8.3) | 0.6241 |
| p-interaction |  | 0.9125 |  | 0.2726 |  | 0.6111 |
| 1,25(OH)_2_D |  |  |  |  |  |  |
| CKD | -21.5 (-84.0, 40.9) | 0.4906 | -15.8 (-84.5, 53.0) | 0.6452 | - 8.2 (-62.2, 45.7) | 0.7595 |
| No CKD | 18.4 (-3.7, 40.5) | 0.1051 | 80.9 (49.7, 112.2) | <0.0001 | 24.5 (2.8, 46.2) | 0.0280 |
| p-interaction |  | 0.0021 |  | 0.0045 |  | 0.3602 |

Model is adjusted for age, race (white vs. nonwhite), clinic, season of blood draw, physical activity, body mass index, smoking status (ever, vs. never), self-reported history of diabetes mellitus, systolic blood pressure, use of medication containing Vitamin D.

Abbreviations: CI: confidence interval; VMR: vitamin D metabolite ratio; PTH: parathyroid hormone; CTX-I: C-terminal telopeptide of type I collagen; PINP: N-terminal propeptide of type I procollagen; CKD: chronic kidney disease (estimated glomerular filtration rate < 60 mL/min/1.73m^2)^.
